# Machine Learning in Ultrasound Computer-Aided Diagnostic Systems: A Survey

**DOI:** 10.1155/2018/5137904

**Published:** 2018-03-04

**Authors:** Qinghua Huang, Fan Zhang, Xuelong Li

**Affiliations:** ^1^School of Mechanical Engineering and Center for OPTical IMagery Analysis and Learning (OPTIMAL), Northwestern Polytechnical University, Xi'an, Shaanxi 710072, China; ^2^College of Information Engineering, Shenzhen University, Shenzhen 518060, China; ^3^School of Electronic and Information Engineering, South China University of Technology, Guangzhou 510641, China; ^4^Center for OPTical IMagery Analysis and Learning (OPTIMAL), Xi'an Institute of Optics and Precision Mechanics, Chinese Academy of Sciences, Xi'an, Shaanxi 710119, China

## Abstract

The ultrasound imaging is one of the most common schemes to detect diseases in the clinical practice. There are many advantages of ultrasound imaging such as safety, convenience, and low cost. However, reading ultrasound imaging is not easy. To support the diagnosis of clinicians and reduce the load of doctors, many ultrasound computer-aided diagnosis (CAD) systems are proposed. In recent years, the success of deep learning in the image classification and segmentation led to more and more scholars realizing the potential of performance improvement brought by utilizing the deep learning in the ultrasound CAD system. This paper summarized the research which focuses on the ultrasound CAD system utilizing machine learning technology in recent years. This study divided the ultrasound CAD system into two categories. One is the traditional ultrasound CAD system which employed the manmade feature and the other is the deep learning ultrasound CAD system. The major feature and the classifier employed by the traditional ultrasound CAD system are introduced. As for the deep learning ultrasound CAD, newest applications are summarized. This paper will be useful for researchers who focus on the ultrasound CAD system.

## 1. Introduction

For decades, ultrasound image has been extensively applied in the detection of different diseases because of its high safety and high efficiency [1–3], such as the breast cancer, the liver cancer, the gastroenteric disease [4], the cardiovascular diseases [5], spine curvature [6], and the muscle disease [7, 8]. However, it requires years of experience and training to read ultrasound image. The amount of training to be an excellent radiologist is high. In this background, the CAD became a powerful tool to assist radiologists diagnosing. The original CAD system was used to diagnose the breast tumor in the 1960s [9]. The CAD system helps the doctors and radiologists to diagnose from two views. One view is their experience; the other is the view of the computer. The application of CAD system improves the accuracy of diagnosis, reduces the time consumption, and decreases the load of doctors [10].

There are two important aspects of CAD research which are “Detection” and “Diagnosis,” respectively [11]. “Detection” is defined as the technology to locate the lesion region of the image. It aims to reduce the observational burden of medical staffs. “Diagnosis” means the technology to identify the potential diseases. It aims to provide additional support for clinicians. In most of the CAD systems, the “Detection” and “Diagnosis” are associated. In the “Detection” phase, the lesion is segmented from the normal tissues, and in the “Diagnosis” phase, the lesion is evaluated to produce a diagnosis.

**Figure 1:**
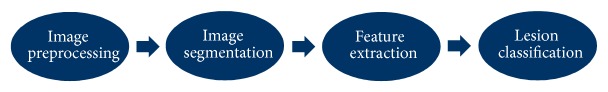
The general flowchart of CAD system.

The ultrasound CAD system also consists of “Detection” and “Diagnosis.” The ultrasound CAD system can be divided into four phases: image preprocessing, image segmentation, feature extraction, and lesion classification.  Figure 1 shows the general flowchart of ultrasound CAD system.

In this article, we present an overview of recent developments in ultrasound CAD to support future studies. There have been many studies which summarized the research of ultrasound CAD [10, 12, 13]. Reference [10] presents a detailed overview of the breast ultrasound CAD research, and [12] presents an overview of liver ultrasound CAD researches. However, both of them ignored many new technologies of the deep learning which is one of the most revolutionary technologies in recent years. In this study, we present an overview of the traditional ultrasound CAD system and the ultrasound CAD system which applies deep learning technology. As for the traditional CAD system, this study focuses on the feature and the classifier. As for the deep learning ultrasound CAD system, the newest applications of deep learning technology in ultrasound CAD system are summarized.

## 2. Traditional Ultrasound CAD System

### 2.1. Feature

As for the traditional ultrasound CAD system, the feature selection and extraction are indispensable steps [29]. The effective features can improve the accuracy and decrease the computational complexity of the system. As for ultrasound CAD system, the collection of data is difficult. If the dimension of features is high and the size of the dataset is small, there will be “curse of dimensionality” occurring [30]. Thus, the selection of features is an important step for traditional ultrasound CAD system. The feature adopted by traditional ultrasound CAD can be divided into four categories: texture, morphologic, model-based, and descriptor features.

#### 2.1.1. Texture

The texture is one of the most common features in the ultrasound CAD system. Texture features can reflect the character of the lesion surface. A few general utilized features are shown as follows.

*Laws Texture Energy (LTE)*. This feature utilizes the local masks to detect the texture types [31]. In general, the size of masks is 5 × 5. The energy of texture is calculated by the local masks and represented by a vector. 

*Contrast of Gray Level Values*. This feature is a measure of local variations in the image. It can be defined as

(1) $$\begin{array}{c} CON=\underset{i,j}{\sum }{\left(\;i-j\;\right)}^{2}P_{d}\left(\;i,j\;\right), \end{array}$$

where *P*_*d*_(*i*, *j*) is the probability of the pixel value (*i*, *j*) lying at distance *d* in the image.

*Gray Level Cooccurrence Matrix (GLCM)*. GLCM reflects the distribution of cooccurring pixel grayscale values at a given offset. GLCM is a common feature in CAD system. The methods in [32, 33] have utilized GLCM to extract the texture features for breast tumor classification. The GLCM can be defined as

(2) $$\begin{array}{c} COR=\frac{\sum ijP_{d}\left(i,j\right)-m_{x}m_{y}}{\sqrt{S_{x}^{2}S_{y}^{2}}}, \end{array}$$

where *m*_*x*_, *m*_*y*_, *S*_*x*_^2^, and *S*_*y*_^2^ are defined as

(3) mx=∑ii∑jPdi,j,my=∑jj∑iPdi,j,Sx2=∑ii∑jPdi,j−mx2,Sy2=∑jj∑iPdi,j−my2.

*Local Binary Pattern (LBP)*. LBP is proposed by T. Ojala, M. Pietikäinen, and D. Harwood. It can reflect the local texture of ultrasound image. The LBP is defined in a 3 × 3 neighborhood. The center of the neighborhood is taken as the threshold. The other 8 gray values are compared to the threshold. If the value is larger than the threshold, that pixel will be marked by 1; otherwise, it will be marked by 0. In this approach, every 3 × 3 neighborhood will be transformed into an 8-bit binary number [34]. LBP possesses the rotation invariance and gray scale invariance. 

*Wavelet Features*. This feature is derived from the wavelet transform of the ultrasound image. The wavelet transform is a generally used method in ultrasound image processing. The method in [21] utilized the wavelet packet transform to extract texture feature for the liver disease classification.

#### 2.1.2. Morphology

Compared with the texture feature, the morphologic feature is more focused on the lesion. We summarized some common morphologic features as follows. 

*Spiculation*. This feature reflects the smoothness of lesion margin. Reference [35] proposed a method to measure the speculation, which defined the spiculation as the ratio of low-frequency area to high-frequency area. This value is proportional to the possibility of the tumor being malignant.

*Depth-to-Width Ratio*. Depth-to-width ratio is an active feature for the classification of many tumors, which has been widely employed by many studies [1, 36]. The depth is defined as the largest difference between the *y*-axis values of two points on the margin of the tumor. The width means the largest difference between the *x*-axis values of two points on the margin of the tumor. As for the malignant tumor, the depth-to-width ratio is usually larger than 1, and the ratio of benign tumor is usually smaller than 1. 

**Figure 2:**
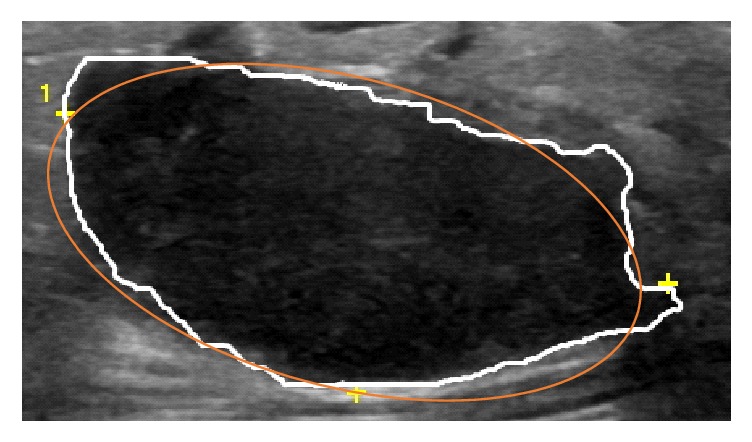
The equivalent ellipse (orange line) of a benign breast lesion.

*Elliptic-Normalized Circumference (ENC)*. ENC is the circumference ratio of the equivalent ellipse of the tumor which is defined as the ratio of the circumference of the ellipse to its diameter [3].  Figure 2 shows an example of equivalent ellipse of a benign breast lesion. 

*Elliptic-Normalized Skeleton (ENS)*. ENS is the number of skeleton points which are normalized by the perimeter of the equivalent ellipse. The larger the ENS is, the higher the possibility of malignancy is [3]. 

*Long Axis-to-Short Axis Ratio (L : S)*. This feature is defined as the ratio of long axis to short axis. The long axis is the major axis of the equivalent ellipse, and the short axis is the minor axis of the ellipse [3].

#### 2.1.3. The Feature Based on Statistical Model of the Backscattered Echo

The model-based feature is one of the unique features of ultrasound images. It reflects the character of the backscattered echo from tissues. Scholars utilized different models to simulate the echo of backscatter. The parameters of these models are employed as tools to classify the tumors. 

*Nakagami Model-Based Features*. Nakagami model is one of the most common models of backscattered echo, which can be utilized to simulate different backscattered distributions. The parameter of Nakagami model is defined by the statistics of the backscattered echoes. The authors in [37] have attempted to utilize the Nakagami parameter as a feature to classify the breast lesion.

*K-Distribution Model-Based Features*. The feature based on *K*-distribution model is also widely used in ultrasound CAD system. Reference [38] utilized the parameter of log-compressed *K* distribution to classify the breast tumor. The experiment in [38] compared the performance of the method employing *K*-*α* feature to the method without *K*-*α* feature. The result shows that the performance of method utilizing *K*-*α* feature is higher than the method without *K*-*α* feature.

#### 2.1.4. Descriptor Features

The descriptor feature is usually summarized from the experience of clinicians. As for different applications, the descriptor feature is different. For example, as for the breast tumor, most of the descriptor features come from the breast imaging reporting and data system (BI-RADS) lexicon. But as for the thyroid nodules, most of the descriptors are attributes in thyroid imaging reporting and data system (TI-RADS) lexicon. 

*Shape (Round, Oval, or Irregular)*. Shape is a universal descriptor feature for classification of many tumors [2]. The regular shape like round and oval usually means that the tumor is benign. The shape of malignant tumor is always irregular. 

*Calcifications (Absent or Present)*. In general, there are more calcifications or microcalcifications in malignant tumor than in benign tumor. 

*Posterior Shadow or Posterior Echo*. The posterior shadow or posterior echo reflects the characteristic of the posterior region of the tumor, where gray value is smaller than the region of the surrounding. 

*Echo Characteristic*. This feature reflects the model of echo in the ultrasound image including hypoechoic, isoechoic, hyperechoic, and complex. The echo signal of different tissues shows different characteristic in the ultrasound image [39].

### 2.2. Classifiers

Most of ultrasound CAD systems are designed to classify the lesion such as the breast tumor, liver fibrosis, and thyroid nodules. The classifier is one of the most important parts in the lesion classification. After the selection and extraction of features, many classifiers are adopted to classify the ultrasound images. This section introduced the major classifiers employed by the ultrasound CAD system.

#### 2.2.1. Linear Classifier

Linear discrimination analysis (LDA) [40] and logistic regression (LOGREG) [41] are two of the most widely used linear classifiers in the ultrasound CAD system. LDA is proposed by Fisher and is extensively used in medical image analysis [32, 42]. It aims to find the best linear combination of the features to divide the data into several categories. LOGREG is proposed by David Cox. It is a regression method which takes the feature as the argument and takes the category as the dependent variable. Both of the LDA and LOGREG are widely applied in medical field [43, 44]. However, the performance of the linear classifier is limited by the distribution of data. If the data is nonlinearly separable, the performance of linear classifier will be unsatisfactory.

#### 2.2.2. Bayesian Classifier

The Bayesian classifier is one of the most frequently used methods in the machine learning field. It can utilize the prior information of data to estimate the posterior information. The most famous Bayesian classifier is the Naïve Bayesian Classifier (NBC). NBC is based on the Bayesian theorem. It hypothesizes that the feature of samples is conditionally independent. There are only a few parameters of NBC which are required for estimation through the statistics of samples. Due to the advantage of insensitivity to data, NBC is widely applied in social information analysis and medical field. Reference [45] utilized the NBC as a classifier to distinguish the cardiovascular US images. The accuracy of the method reached 96.59% [45].

#### 2.2.3. Support Vector Machine

The support vector machine (SVM) is a method in statistics and computer science to analyze data and recognize pattern. It is a supervised learning method which can be applied in both of classification and regression. The target of SVM is to build a hyperplane to divide the sample into different categories [46]. It utilized the kernel functions to map the original data into the higher dimensional space to find the decision hyperplane. SVM is widely applied in the analysis of ultrasound images [15, 47–49]. SVM can perform well in both of small dataset and large dataset. However, as the size of dataset increases, the complexity of SVM also grows. Meanwhile, the choice of kernel function also influences the performance of SVM.

#### 2.2.4. Decision Tree

The decision tree is an effective algorithm for classification of ultrasound images [25, 50]. It can learn a classification rule from disorder data. Decision trees algorithm adopts the divide-and-conquer strategy to divide search space of problem into several subsets. The structure of decision tree is a flowchart. From top to bottom, every node calculates the feature value of input sample to decide which node to go to next. In leaf nodes, the final result of classification is given [51]. When the size of data is small and the feature value is not diverse, the construction of decision tree is simple and fast. However, if the size of data is large and the feature value is various, the complexity of decision tree algorithm will be huge.

#### 2.2.5. Artificial Neural Network

Artificial neural network (ANN) is the machine learning model which is designed according to the human nervous system. In general, the architecture of ANN can be divided into three layers: the input layer, the hidden layer, and the output layer. The layer consists of the neuron. The number of the hidden layers and the number of the neurons in each layer are flexible. One of the most famous ANN is the back-propagation neural network (BPNN) [52]. BPNN is a feed-forward ANN with supervised learning process. It is widely used in the medical image analysis [53–55]. The train of ANN is a self-adaptive process. If the architecture is complex, it will take plenty of time to train the network.

#### 2.2.6. AdaBoost

AdaBoost is one of the most popularly used ensemble methods proposed and has the ability to improve the classification accuracy by integrating multiple weak classifiers. AdaBoost method generates a series of weak classifiers firstly and builds a powerful classifier through weighted majority voting of the classes predicted by weak classifiers. Reference [38] utilized the multiclass AdaBoost to distinguish carcinomas, fibro adenomas, and cysts.

## 3. Ultrasound CAD System with Deep Learning Technology

In 2006, the professor of the University of Toronto, Hinton, and his student published the paper which utilized the neural network to reduce the dimensionality of data [56]. This paper is widely regarded as the beginning of the research in deep learning. In the following years, deep learning was extensively applied in many fields, such as image recognition, semantic analysis, and disease detection. The ultrasound CAD system is always a highly anticipated field where the deep learning can be applied. Many scholars have attempted to utilize the deep learning to assist the clinician.

The largest change from the traditional ultrasound CAD to deep learning ultrasound CAD is that the feature employed by deep learning ultrasound CAD system is not artificial. In the traditional ultrasound CAD system, most of the features are human-crafted, such as gray features and texture features. However, with the development of deep learning, the researchers noted that the feature extracted by the deep neural network is sometimes more effective than the feature designed by the human.

In this section, the newest applications of deep learning on the ultrasound CAD system are introduced. The major application field includes the breast lesion diagnosis, the liver lesion diagnosis, the fetal ultrasound standard plane detection, the thyroid nodule diagnosis, and the carotid ultrasound image classification.

### 3.1. The Breast Lesion Diagnosis

The breast tumor is one of the most common cancers for women. Thousands of women suffer from breast tumor all over the world. The early detection can decrease the death rate of the breast cancer significantly [57]. The ultrasonography is a safe and convenient scheme to detect the early breast lesion [58]. To support the clinician in diagnosis, many scholars attempted to utilize the deep learning technology to classify the breast lesion. Han et al. utilized the GoogLeNet to classify the breast image and the accuracy reached 90% [16]. They employed 4254 benign samples and 3154 malignant samples to train the deep neural network. The sufficient data support the GoogLeNet to reach an acceptable performance. However, more researchers cannot acquire enough data like Han et al. [16]. Most of them employed other deep learning methods to classify the breast lesion. Zhang et al. utilized the point-wise gated Boltzmann machine (PGBM) to extract the feature from shear-wave elastography (SWE) to classify the breast tumor [17]. The deep learning feature reached 93.4% accuracy. Cheng et al. utilized stacked denoising autoencoder (SDAE) technology to encode the ultrasound image and employed the softmax layer to classify the breast lesion [18]. Shi et al. employed the deep polynomial network to extract the textural feature from the ultrasound image and reach the accuracy of 90.40% [19].

The deep learning technology is widely applied in the breast ultrasound image. However, most of the studies are limited by the number of samples. Methods adopted by these studies usually utilized the deep learning technology as a tool to generate the representation of images. Only [16] utilized the convolutional neural network (CNN) like GoogLeNet to classify the ultrasound image directly.

### 3.2. The Liver Lesion Diagnosis

The liver disease has been a menace to humans for a long time. The incidence and mortality of the liver disease grow yearly. The ultrasonography is one of the most common techniques to detect the liver disease. Many researchers have attempted to employ deep learning technology to support the doctor diagnosis by liver ultrasound image. Reference [22] utilized the sparse autoencoder to acquire the representation of the liver ultrasound image and utilized the softmax layer to distinguish different focal liver diseases. Compared with support vector machines method, the method proposed in [22] reaches higher accuracy.

Liver fibrosis classification is also a high profile field of research. Meng et al. utilized the VGGNet and fully connected network (FCN) to differentiate the level of liver fibrosis [23]. To address the shortage of samples, Meng et al. employed the transfer learning (TL) technology. Meng et al. divided the liver fibrosis level into three phases: normal, early stage fibrosis (S1–S3), and late-stage fibrosis (S4). The accuracy of their method reached 93.90%. Similarly to Meng et al., Liu et al. utilized deep learning technology to diagnose the cirrhosis [24]. In the study of Liu et al., CNN is employed as a tool to generate features from ultrasound images. Liu et al. adopted the SVM as the classifier to distinguish the normal liver and the diseased liver, and the accuracy of the proposed method reached 96.8% which is much higher than the accuracy of low-level features.

The deep learning is a powerful tool to detect the liver diseases from ultrasound liver images. According to the experiment result of [23, 24], the application of deep learning technology can significantly improve the accuracy of liver diseases diagnosis.

### 3.3. The Fetal Ultrasound Standard Plane Detection

The ultrasound imaging is one of the most common technologies in the prenatal examination for being economic and safe. Standard plane selection is one of the necessary phases in the ultrasound examination [59, 60]. The clinician can estimate subsequent biometric information of fetus from the fetal ultrasound standard plane. Many scholars have attempted to utilize the machine learning technology to detect the fetal ultrasound standard plane automatically. With the popularity of deep learning, the researchers began to utilize the deep learning to distinguish the fetal ultrasound plane. The fetal facial standard plane is one type of the fetal ultrasound standard plane. From the fetal facial standard plane, the doctor can measure the biparietal diameter of the fetus and detect the malformation. Yu et al. employed the CNN to classify the fetal ultrasound plane. Their method reached the accuracy of 93.03% which is much higher than the accuracy of the traditional method [61]. However, the time consumption of training which often takes more than 80 hours for the method is very expensive.

The study of Yu et al. focuses on one type of the fetal ultrasound standard plane. Their method cannot distinguish other types of the fetal ultrasound standard plane. Chen et al. proposed a deep learning framework which can detect different types of the fetal ultrasound standard plane [62]. Chen et al. employed the CNN and long short-term memory (LSTM) model to classify the fetal abdominal standard plane (FASP), the fetal face axial standard plane (FFASP), and the fetal four-chamber view standard plane (FFVSP). The CNN is responsible for extracting features from ROI images, and the LSTM model is responsible for the classification. Although the method proposed by Chen et al. [62] can classify different types of the fetal ultrasound standard, its performance is slightly lower than the method in [61]. The accuracy of FASP is 90.80%, the accuracy of FFASP is 86.70%, and the accuracy of FFVSP is 86.70%.

Besides the fetal ultrasound standard plane, the deep learning was also applied in the detection of fetal neurosonographic diagnostic plane. The fetal neurosonographic diagnostic plane can help the clinician to estimate the growth of fetal head and detect the serious central nervous system anomalies. Reference [63] proposed a method which employed CNN to detect the fetal neurosonographic diagnostic plane. The experiment result shows that the method in [63] has a similar accuracy to a specialist's performance.

The fetal ultrasound standard plane detection is one of the research fields where the deep learning can be applied. Unlike the breast lesions diagnosis and liver diseases diagnosis, the collection of the fetal ultrasound standard plane samples is more convenient. There are sufficient samples which can be utilized to train the deep learning network.

### 3.4. The Thyroid Nodule Diagnosis

The thyroid nodule is a common disease upon a world scale. The ultrasound imaging is a widely employed scheme to detect the thyroid nodule. To support the doctor to diagnose the thyroid nodule, many CAD systems were proposed. With the breakthrough of deep learning, many scholars focus on the method which employs the deep learning to classify the thyroid nodule. Chi et al. employed the GoogLeNet to classify the thyroid nodule [27]. To address the shortage of data, Chi et al. utilized the Deep Learning Caffe library [64] to fine-tune the GoogLeNet. The accuracy of their method reached 99.13%. Reference [28] presented a method which employed cascade CNN to detect and classify the thyroid nodule. The cascade CNN in [28] includes two CNNs. The first CNN was responsible for the segmentation of thyroid nodules, and the second CNN was utilized to classify the thyroid nodules. The experiment shows that the cascade CNN method outperforms other traditional machine learning methods.

The deep learning can improve the performance of thyroid nodule diagnosis significantly. However, the time consumption of train the deep learning network is also enormous. In [28], the training time of cascade CNN which is accelerated by GPU is more than 106 hours. The more complex the model is, the larger the cost of training is.

### 3.5. The Carotid Ultrasound Image Classification

The mortality of cardiovascular diseases increases yearly. The atherosclerotic plaque is the major reason of cardiovascular diseases. In the early detection of atherosclerosis, the intima-media thickness (IMT) of the carotid artery is an important indicator. IMT is the distance between the lumen-intima interface (LII) and the media-adventitia interface (MAI). The doctor usually utilized the ultrasound image to measure the IMT. To support the diagnosis of doctors, the researcher has attempted to utilize the deep learning to acquire the IMT automatically. Reference [65] utilized the autoencoder to segment LII and MAI. The IMT was acquired by calculating the distance between two levels. The error of the method in [65] is much smaller than traditional methods.

Besides the calculation of IMT, the deep learning method also is applied to detect the composition of plaque. Reference [66] utilized CNN to classify different tissues of plaque including lipid core, fibrous tissue, and calcified tissue. The experiment shows that the classification accuracy of CNN is much better than SVM.

### 3.6. Other Applications

Besides the application mentioned above, there are some other applications of deep learning on the ultrasound CAD system. The study in [67] applied CNN to classify the type of myositis including inclusion body myositis (IBM), polymyositis (PM), and dermatomyositis (DM). Reference [67] compared the performances of CNN and random forests. The accuracy of CNN for normal versus affected tissues (DM, PM, and IBM) reached 76.2% which is 3.9% higher than this value of random forests. Hetherington et al. designed a spine level identification system employing CNN [68]. The system can accurately detect the vertebral level so that the anesthesiologist can find the right site to inject the anaesthetic. Cheng and Malhi utilized CNN to classify the abdominal ultrasound images [69]. In the paper, Cheng and Malhi divided the abdominal ultrasound images into 11 categories including liver left longitudinal, liver left transverse, liver right longitudinal, liver right transverse, spleen, pancreas, kidney left longitudinal, kidney left transverse, kidney right longitudinal, kidney right transverse, and gallbladder. The mean accuracy of classification reached 77.9%.

## 4. Performance Summary

In this section, we summarized the performance of various techniques in the different application fields.  Table 1 shows the performance of breast ultrasound CAD system.  Table 2 shows the performance of liver ultrasound CAD system.  Table 3 shows the performance of thyroid ultrasound CAD system.

## 5. Discussion and Conclusions

In this study, we summarized the literature about the ultrasound CAD system. This study divided the ultrasound CAD system into two categories. One is the traditional ultrasound CAD system which employs the manmade feature. The major feature and major classifier adopted by the traditional ultrasound CAD system are introduced. Another category is the deep learning ultrasound CAD system which employs the deep neural network to extract features and classify them. The newest applications of deep learning on the ultrasound CAD system were summarized.

As for the traditional ultrasound CAD system, the selection of feature impacts the performance of final diagnosis. The common feature employed by traditional ultrasound CAD system can be divided into four categories: textural features, morphologic features, model-based features, and descriptor features. The textural feature is one of the earliest adopted features in the ultrasound CAD system. TEM, GLDS, GLCM, and other textual features are widely applied in the classification of liver diseases and breast lesions. The morphologic feature is a powerful feature in the traditional ultrasound CAD system. It contains the prior knowledge of clinicians. Morphologic features like spiculation and depth-to-width ratio are designed according to the experience of clinicians. These features are extracted from the ultrasound image automatically and are extensively adopted in the ultrasound CAD system. Model-based features are based on the backscattered echo of ultrasound images. Nakagami model-based features and *K*-distribution model-based features are two common model-based features. The descriptor feature is usually summarized from clinical experience. As for the different application, the descriptor feature is different.

The classifiers employed by traditional ultrasound CAD system are divided into 6 categories: linear classifier, Bayesian method, SVM, decision tree, ANN, and AdaBoost. Both of the linear classifier and Bayesian method are common classifiers in the machine learning field. These two classifiers are convenient to use. However, the performance of them is not stable on all of the data. The decision tree is also a simple algorithm, and the complexity of it is low. The SVM is a powerful classifier. It can perform well even in the small dataset. As for ANN, there is no certain rule in the design of ANN. It is flexible and widely applicable. The AdaBoost can integrate the output of weak classifiers to get a robust classification result.

The largest difference between the deep learning ultrasound CAD system and traditional ultrasound CAD system is the approach of extracting features. In the traditional ultrasound CAD system, the feature is designed by the human. But in the deep learning ultrasound CAD system, the feature is extracted by deep learning network automatically. This paper introduced the newest application of deep learning on the ultrasound CAD system. The application field includes the breast lesion diagnosis, the liver lesion diagnosis, the fetal ultrasound standard plane detection, the thyroid nodule diagnosis, and the carotid ultrasound image classification.

This study summarized the performance of ultrasound CAD in three fields including breast tumor classification, liver diseases, and thyroid nodule diagnosis. It can be seen that the dataset employed by these studies is different. There are huge differences in the size and the modality of the dataset employed by different methods. It is hard to fairly evaluate the performance of different methods utilizing different datasets. The construction of standard dataset for different ultrasound CAD applications is an important task in further studies.

On the other hand, the collection of ultrasound data is also a problem. Deep learning methods require plenty of samples to train the network. However, the size of the dataset employed by most of the studies mentioned above is still small. The shortage of ultrasound samples is one of the obstacles in the way of applying deep learning.

## Figures and Tables

**Table 1:** The performance summary of breast ultrasound CAD system.

Reference	Dataset	Features	Classifiers	Performance
[14]	88 benign 90 malignant	Textural features + morphologic features	ANN (BPNN)	Accuracy: 95.86% Sensitivity: 95.14% Specificity: 96.58%

[15]	70 benign 50 malignant	Textural features + morphologic features	SVM	Accuracy: 95.83% Sensitivity: 96% Specificity: 95.71%

[16]	4254 benign 3154 malignant	GoogLeNet		Accuracy: 91.23% Sensitivity: 84.29% Specificity: 96.07%

[17]	135 benign 92 malignant	Boltzmann machine		Accuracy: 93.4% Sensitivity: 88.6% Specificity: 97.1%

[18]	275 benign 245 malignant	Stacked denoising Autoencoder (SDAE)		Accuracy: 82.4% Sensitivity: 78.7% Specificity: 85.7%

[19]	100 benign 100 malignant	Deep polynomial network	SVM	Accuracy: 92.40% Sensitivity: 92.67% Specificity: 91.36%

**Table 2:** The performance summary of liver ultrasound CAD system.

Reference	Dataset	Features	Classifiers	Performance
[20]	50 normal 50 fatty liver disease (FLD)	Textural features	ANN	Accuracy: 98% Sensitivity: 100% Specificity: 96%

[21]	15 normal 16 cirrhotic 25 hepatocellular carcinoma (HCC)	Textural features	SVM	Accuracy: 88.8%

[22]	44 cyst 18 hemangioma 30 HCC 16 normal	Sparse autoencoder		Accuracy: 90.50% Sensitivity: 91.60% Specificity: 88.50%

[23]	79 normal 89 early-stage fibrosis 111 late-stage fibrosis	VGGNet	FCN	Accuracy: 93.90% Sensitivity: 88.6% Specificity: 97.1%

[24]	47 cirrhosis 44 normal	CNN	SVM	Accuracy: 86.9%

**Table 3:** The performance summary of thyroid ultrasound CAD system.

Reference	Dataset	Features	Classifiers	Performance
[25]	48 benign 223 malignant	Textural features	Decision tree: C4.5	Accuracy: 94.3%

[26]	10 benign 10 malignant	Textural features	AdaBoost	Accuracy: 100% Sensitivity: 100% Specificity: 100%

[27]	71 benign 357 malignant	GoogLeNet		Accuracy: 99.13% Sensitivity: 99.70% Specificity: 95.80%

[28]	465 normal 9957 thyroid nodular lesions	CNN (15 convolutional layers)	CNN (4 convolutional layers)	AUC: 0.986
